# Detecting white adipose tissue browning in mice with in vivo R_2_∗ mapping at 9.4T MRI

**DOI:** 10.1016/j.jlr.2024.100735

**Published:** 2024-12-19

**Authors:** Qiaoling Zhong, Hongsheng Liu, Yanqiu Feng, Xiuwei Jiao, Yuanbo Yang, Daming Zhang, Qian Wang, Zoheb Ahasan, Andrew Z. Li, Chong Wee Liew, Zimeng Cai, Zaiyi Liu, Kejia Cai

**Affiliations:** 1 Department of Radiology, Guangzhou Women and Children's Medical Center, Guangzhou Medical University, Guangzhou, China; 2 School of Biomedical Engineering, Southern Medical University, Guangzhou, China; 3 Guangdong Provincial Key Laboratory of Medical Image Processing & Guangdong Province Engineering Laboratory for Medical Imaging and Diagnostic Technology, Southern Medical University, Guangzhou, China; 4 Guangdong-Hong Kong-Macao Greater Bay Area Center for Brain Science and Brain-Inspired Intelligence & Key Laboratory of Mental Health of the Ministry of Education, Southern Medical University, Guangzhou, China; 5 Department of Radiology, Shunde Hospital, Southern Medical University (The First People's Hospital of Shunde, Foshan), Foshan, China; 6 Department of Radiology, Guangdong Provincial People’s Hospital (Guangdong Academy of Medical Sciences), Southern Medical University, Guangzhou, China; 7 Guangdong Provincial Key Laboratory of Artificial Intelligence in Medical Image Analysis and Application, Guangzhou, China; 8 State Key Laboratory of Bioactive Molecules and Draggability Assessment, The Biomedical Translational Research Institute, Health Science Center (School of Medicine), Jinan University, Guangzhou, China; 9 Key Laboratory of Viral Pathogenesis & Infection Prevention and Control (Jinan University), Ministry of Education, Guangzhou, China; 10 Department of Radiology, University of Illinois at Chicago, Chicago, IL, USA; 11 Department of Biomedical Engineering, University of Illinois at Chicago, Chicago, IL, USA; 12 New Trier Township High School, Winnetka, IL, USA; 13 Physiology and Biophysics Department, University of Illinois at Chicago, Chicago, IL, USA

**Keywords:** white adipose tissue, browning, R_2_∗, iron, UCP1

## Abstract

White adipose tissue (WAT) browning is considered a promising strategy to combat obesity and related metabolic diseases. Currently, fat-water fraction (FWF) has been used as a marker for the loss of lipids associated with WAT browning. However, FWF may not be sensitive to metabolic changes and cannot specifically reflect iron-regulated metabolism during browning. Here, we report a noninvasive preclinical imaging approach based on iron content detected by R_2_∗ mapping to assess in vivo WAT browning in mice. In this study, we investigated the browning of inguinal white adipose tissue (iWAT) induced by long-term CL-316,243 (CL) drug stimulation in mice. We quantified the changes in R_2_∗, FWF, uncoupling protein 1 (UCP1) expression, and iron content. The iWAT of all mice was dissected for H&E staining and immunohistochemistry for the absorbance of UCP1 and iron content. In in vivo experiments, a significant increase in R_2_∗ and a decrease in FWF were observed in iWAT after 7 days of CL administration compared with the saline-treated and the baseline groups. Accordingly, in ex vivo experiments, UCP1 expression and the total iron content in iWAT significantly increased after 7 days of CL stimulation. By pooling all mice data, the UCP1 expression level of iWAT and iron content was found to be highly correlated with R_2_∗ and inversely correlated with FWF. Taken together, R_2_∗ may serve as a potential imaging biomarker for assessing WAT browning, which provides a new diagnostic and therapeutic evaluation tool for metabolic diseases.

The World Health Organization has shown that overweight and obesity are one of the major risks of premature death (1). According to the World Obesity Atlas 2023 report, by 2035, over half of the global population is projected to be overweight or obese. Obesity not only affects people's appearance and mobility but also greatly increases the risks of type 2 diabetes, cardiovascular disease, metabolic syndrome, malignant tumor, and other chronic diseases, resulting in higher morbidity and mortality (2, 3).

Adipose tissue in mammals is primarily categorized into white adipose tissue (WAT), brown adipose tissue (BAT), and beige adipose tissue. WAT, a nonthermogenic fat, is mainly composed of single-cell adipocytes that store excess energy in the form of triglycerides. BAT and beige fat, on the other hand, are thermogenic fats characterized by multicompartment lipid droplets, high mitochondrial content, and the presence of uncoupling protein 1 (UCP1). Beige adipose tissue is unique, as it originates from a white adipocyte precursor but functions similarly to BAT, making it an intermediate form between WAT and BAT. Both BAT and beige fat promote energy expenditure through UCP1-mediated nonshivering thermogenesis, playing a role in combating obesity and related metabolic disorders (4, 5, 6). WAT can be stimulated by prolonged cold exposure or β_3_-adrenergic receptor (ADRB3) agonists to convert into adipocytes with a brown-like phenotype, a process known as “WAT browning” or “beiging” (7, 8). Selective induction of WAT browning is considered one of the most promising strategies to combat obesity and related metabolic diseases. In recent years, multiple studies have been focused on exploring different strategies for WAT browning (9, 10, 11). In the meantime, it is highly desirable for developing and verifying sensitive, noninvasive, and in vivo imaging methods for the detection of WAT browning or beiging.

During the WAT browning process, adipocytes exhibit a high degree of plasticity, characterized by an increase in multilocular small lipid droplets and mitochondrial biogenesis, among other changes (12, 13). As a result, mitochondrial content can serve as an indicator of beige fat formation to some extent. Mitochondria are abundant in iron-containing enzymes and iron–sulfur cluster proteins, which possess high redox activity. It has been reported that mitochondria hold approximately 20%–50% of the cell's total iron content, primarily contributing to the synthesis of heme and iron–sulfur clusters within the mitochondrial matrix (14, 15). Compared with WAT, beige adipose tissue contains a higher amount of iron and mitochondria, providing a significant clue for assessing beige adipogenesis. Furthermore, the browning of WAT raises its iron demand and utilization to support the enhanced thermogenic capacity (16, 17). As a result, the extent of WAT browning may be roughly measured by its iron content. This approach may offer a novel strategy for monitoring and regulating WAT metabolism to combat obesity and metabolic diseases.

At present, the Dixon MRI technique is commonly used for fat quantification, which can quantify the fat-water fraction (FWF) and R_2_∗ simultaneously. FWF is calculated based on the proportion of fat and water signals within the tissue, which reflects the lipid oxidation of BAT and beige adipose tissue indirectly (18, 19). However, FWF cannot directly reflect fat metabolism. In contrast, R_2_∗ can reflect the iron content level of tissues and can quantify the metabolism of tissues to a certain extent, especially for the metabolically active and iron-rich tissues such as BAT (20). During WAT browning, many mitochondria are generated, and the demand and utilization of iron increases, leading to an increase in the total iron content of adipose tissue (21). For that reason, variation of R_2_∗ could reflect the change of iron content in adipose tissue during this process.

However, whether R_2_∗ can effectively monitor WAT browning is still unknown. Therefore, this study aimed to evaluate the feasibility of R_2_∗ in detecting WAT browning. In this study, inguinal white adipose tissue (iWAT) was imaged to observe the browning process of WAT. The changes of R_2_∗ and FWF of WAT during long-term β_3_ adrenergic receptor agonist CL-316,243 (CL) stimulation are quantified in vivo. Additionally, we observed the changes in ex vivo total iron content and UCP1 protein expression level and investigated their correlations with the R_2_∗ and FWF.

## Materials and methods

### Animal protocols

All animal experiments were approved by the Institutional Animal Use and Care Committee of Guangdong Provincial People’s Hospital. Ten-week-old C57BL/6 mice were kept at room temperature at 23°C with a 12:12 h light and dark cycle and free access to food and water. Eighteen mice were randomly assigned to three groups: baseline group, saline group, and CL group. Mice in the baseline group were not treated, and the CL and saline groups were injected with CL (1 mg/kg body weight, Sigma-Aldrich) or saline daily for 7 days, respectively.

### MRI

MRI of mice was performed with a 9.4T animal scanner (Bruker Biospec, Billerica, MA). Mice were anesthetized with isoflurane (induced with 3% and maintained with 1.5%) to keep the breathing rate at 60 times/min. Respiratory gating was used to trigger image acquisition to reduce motion artifacts. A circulating water heating pad was used to maintain the mice's body temperature at 37.5 ± 0.2°C.

Rapid acquisition with relaxation enhancement sequence-based T_2_-weighted anatomical images were acquired covering the iWAT with the following parameters: repetition time = 6,000 ms; echo time (TE) = 5 ms; rapid acquisition with relaxation enhancement factor = 12; field of view = 40 × 30 mm^2^; matrix = 200 × 150; slice thickness = 1.0 mm; number of slices = 25; number of averages = 1; and the total acquisition time = 1 min 12 s.

Spoiled gradient echo sequence-based Dixon MRI was acquired for FWF and R_2_∗ imaging with the following parameters: repetition time = 500 ms; TE = 1.47 ms; ΔTE = 1.10 ms; number of TE = 10; receiver bandwidth = 200 kHz; field of view = 40 × 30 mm^2^; matrix = 80 × 60; slice thickness = 1.0 mm; flip angle = 10°; number of slices = 20; number of averages = 3; and the total acquisition time = 2 min. The Dixon MRI was performed with the same spatial position as the T_2_-weighted images.

### Imaging processing

All the postprocessing algorithms used in this study were developed in MATLAB (r2020a; MathWorks, Natick, MA). For Dixon MRI, multiple echo complex images acquired from the spoiled gradient echo sequence were reconstructed from real and imaginary images, and then the water and fat images were estimated according to the iterative least-squares algorithm (IDEAL, iterative decomposition of water and fat with echo asymmetry and least-squares estimation). Voxel-based signals over TEs, S(TE_n_), contain water and fat species that can be expressed as:

(2) $$S\left({TE}_{n}\right)=\left(W+F\sum_{\rho =1}^{P}r_{\rho }e^{-i2\pi △f_{\rho }{TE}_{n}}\right)e^{-i2\pi \psi {TE}_{n}}e^{-{R_{2}}^{*}{TE}_{n}}$$

Here, W and F are the complex intensity of water and fat respectively, $r_{\rho }$ (ρ = 1, …, P) is the contribution of the $\rho_{th}$ fat’s species to the S(TE_n_) when TE = 0, △$f_{\rho }$ is the resonant frequency of the $\rho_{th}$ fat peak relative to the water peak, ψ represents the B_0_ field (in Hertz), at this voxel. The fat spectral model we used for FWF calculation in mice included six fat peaks at 0.9, 1.3, 2.1, 2.75, 4.2, and 5.3 ppm with percentages of the total fat signal of 8.8%, 70%, 12%, 0.6%, 3.9%, and 4.7%, respectively. The FWF was calculated by the following equation:

(3) $$FWF=\frac{F}{F+W}$$

### Imaging analysis

**Fig. 1:**
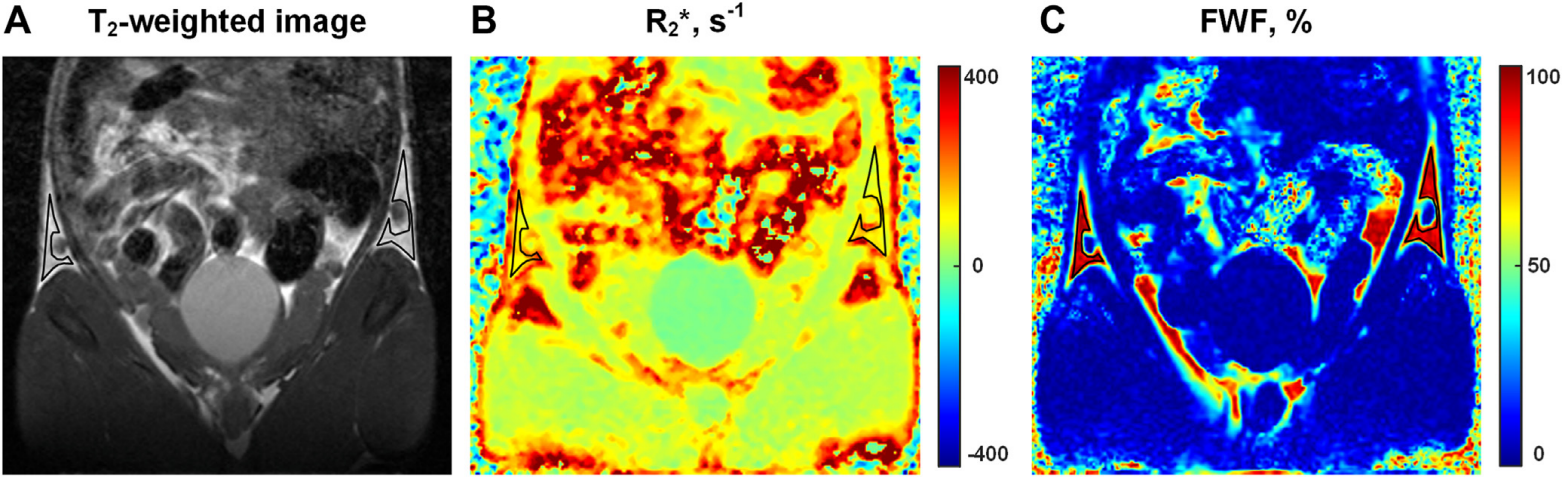
Schematic representation of the ROI delineation of iWAT. The ROI (black outlined) of the iWAT was first outlined on the coronal T_2_-weighted image (A) and then copied to the corresponding R_2_∗ map (B) and FWF map (C). ROI was delineated on both the left and right sides of each slice, avoiding the lymph nodes, blood vessels, skeletal muscles, and air spaces.

The region of interest (ROI) was delineated by radiologists with more than 5 years of adipose tissue MRI experience using ITK-SNAP software. The FWF and R_2_∗ values of iWAT were measured in manually drawn ROIs in reference to the T_2_-weighted images. Based on previous iWAT browning studies (22, 23, 24) and given that cold-induced iWAT browning mainly occurs in the core region of iWAT, which is close to the inguinal lymph node (22, 25), the middle three slices containing the lymph node were selected for ROI delineation. ROI was delineated on both the left and right sides of each slice, avoiding the lymph nodes, blood vessels, skeletal muscles, and air spaces (Fig. 1).

### Histologic assessment and the measurement of iron content of mice iWAT

The iWAT of mice same as indicated in the MRI was dissected, fixed in 10% formalin, and embedded in paraffin. Embedded paraffin samples were cut into 4-μm thick slices for H&E and immunohistochemistry (IHC) staining. Standard H&E staining was performed on rehydrated fat-paraffin sections. For IHC, the rabbit-specific HRP/DAB (ABC) detection IHC Kit (ab64261; Abcam) was used for UCP1 (ab10983; Abcam, 1:1,000 dilution) IHC according to the manufacturer’s instructions. H&E and IHC staining sections were scanned using the Aperio ScanScope XT at 20x magnification, generating a .tif image file per section for each mouse iWAT. The absorbance of the IHC images was analyzed using ImageJ (NIH Image, Bethesda, MD) to evaluate the UCP1 expression level. For iron analysis, iWAT samples were normalized with lysis buffer at 20 μl/mg. The total iron content of iWAT was measured using the Tissue Total Iron Content Colorimetric Assay Kit (E1050; Applygen) according to the manufacturer’s instructions.

### Gene expression analysis

Total RNA was extracted using the TRNzol Universal Reagent (TIANGEN Biotech), and the concentration was measured by the NanoDrop 2000 ultraviolet-visible spectrophotometer (Thermo Fisher Scientific). RT-QPCR and PrimeScript RT Reagent Kit (Takara Bio) were used to reverse transcribe complementary DNA with 1 μg total RNA. The complementary DNA was detected with TB Green Premix EX Taq II kit (Takara Bio) and CFX96 Real-Time PCR assay system (Bio-Rad Laboratories). Using hypoxanthine-guanine phosphoribosyltransferase (Hprt) mRNA as the reference, normalized mRNA expression was calculated by $2^{-\Delta \Delta C_{T}}$ method. The sequence of primers used is as follows: Hprt (forward: TCATTATGCCGAGGATTTG; reverse: GCCTCCCATCTCCTTCAT); Ucp1 (forward: ACTGCCACACCTCCAGTCATT; reverse: CTTTGCCTCACTCAGGATTGG); Dio2 (forward: GCCAACGTAGCTTATGGGGT; reverse: TTCTCCAGCCAACTTCGGAC); Ppargc1α (forward: CCCTGCCATTGTTAAGACC; reverse: TGCTGCTGTTCCTGTTTTC).

### Statistical analysis

GraphPad Prism (version 8.3.0; GraphPad Software, Inc) was used for statistical analysis. All data are presented as mean ± SD unless otherwise stated. Two-tailed Student’s *t*-tests were used for comparisons between the two groups. Pearson’s correlation was used to estimate the relationship between two quantitative variables. *P* < 0.05 was considered statistically significant.

## Results

### The changes of weight and iWAT of mice after repeated CL stimulation

**Fig. 2:**
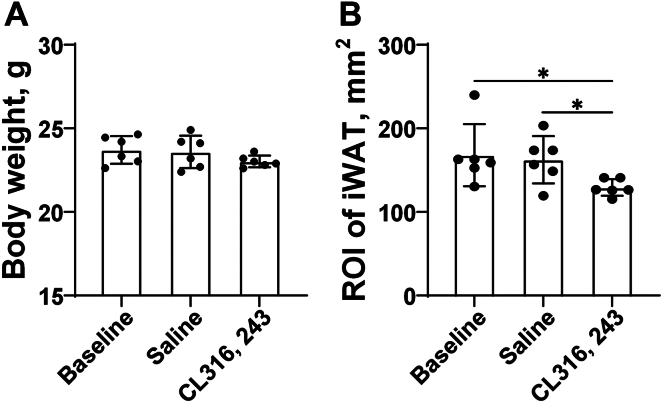
The changes of weight and iWAT of mice after repeated CL stimulation. A: The body weight of mice in baseline, saline, and CL groups. B: The ROI size of iWAT in mice in baseline, saline, and CL groups. Data are presented as mean ± SD. ∗*P* < 0.05.

In Fig. 2A, there was no statistically significant difference in body weight between the three groups (*P* > 0.05). As shown in Fig. 2B, the ROI size of iWAT in the CL group was smaller than that in the baseline group and the saline group (baseline vs. CL: *P* < 0.05; saline vs. CL: *P* < 0.05).

### The R_2_∗ values of iWAT increased significantly after repeated CL stimulation

**Fig. 3:**
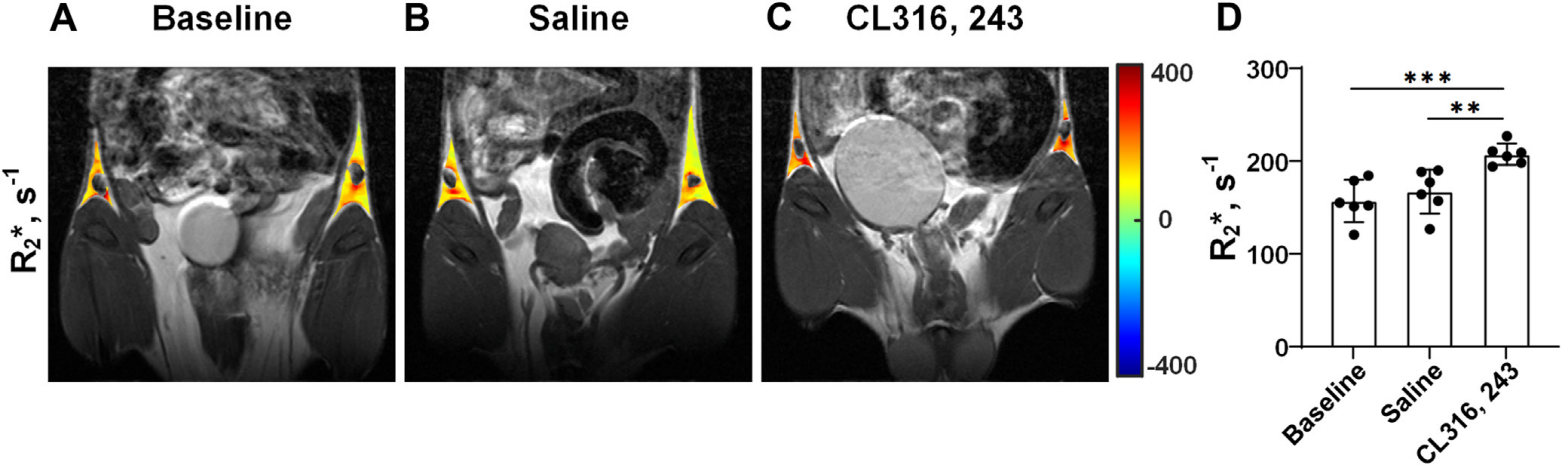
Repeated CL treatment increased the R_2_∗ of iWAT. Representative R_2_∗ images of the iWAT of mice from the baseline (A), saline (B), and CL groups (C), respectively. D: Comparison of R_2_∗ values of iWAT of mice among the baseline, saline, and CL groups. Data are presented as mean ± SD. ∗∗*P* < 0.01, ∗∗∗*P* < 0.001.

As shown in Fig. 3, the R_2_∗ values of iWAT in baseline, saline, and CL groups were 157.1 ± 22.9 s^−1^, 167.2 ± 23.8 s^−1^, and 207.3 ± 11.5 s^−1^, respectively. Compared with the baseline and saline groups, the R_2_∗ value of iWAT in the CL group was significantly increased (baseline vs. CL: *P* < 0.001; saline vs. CL: *P* < 0.01). There was no statistical difference in the R_2_∗ value of iWAT between baseline and saline groups (*P* = 0.47).

### The FWF of iWAT decreased significantly after repeated CL stimulation

**Fig. 4:**
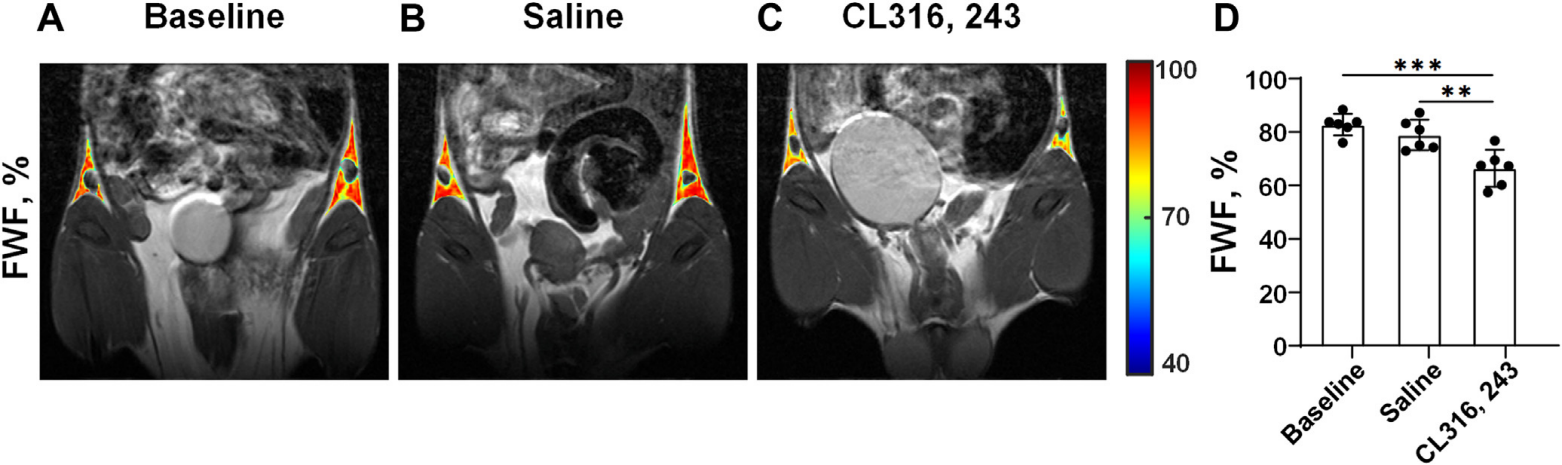
Repeated CL treatment decreased the FWF of iWAT. Representative FWF images of the iWAT of mice in the baseline (A), saline (B), and CL groups (C), respectively. D: Comparison of FWF of iWAT of mice among the baseline, saline, and CL groups. Data are presented as mean ± SD. ∗∗*P* < 0.01, ∗∗∗*P* < 0.001.

As shown in Fig. 4, the FWF of iWAT at baseline, saline, and CL groups were 82.8% ± 4.0%, 79.0% ± 5.7%, and 66.4% ± 6.9%, respectively. Compared with the baseline and saline groups, the FWF of iWAT significantly decreased after 7 days of CL injection (baseline vs. CL: *P* < 0.001; saline vs. CL: *P* < 0.01). There was no statistical difference in the FWF of iWAT between the baseline group and the saline group (*P* = 0.21).

### Histology staining detects the browning of iWAT

**Fig. 5:**
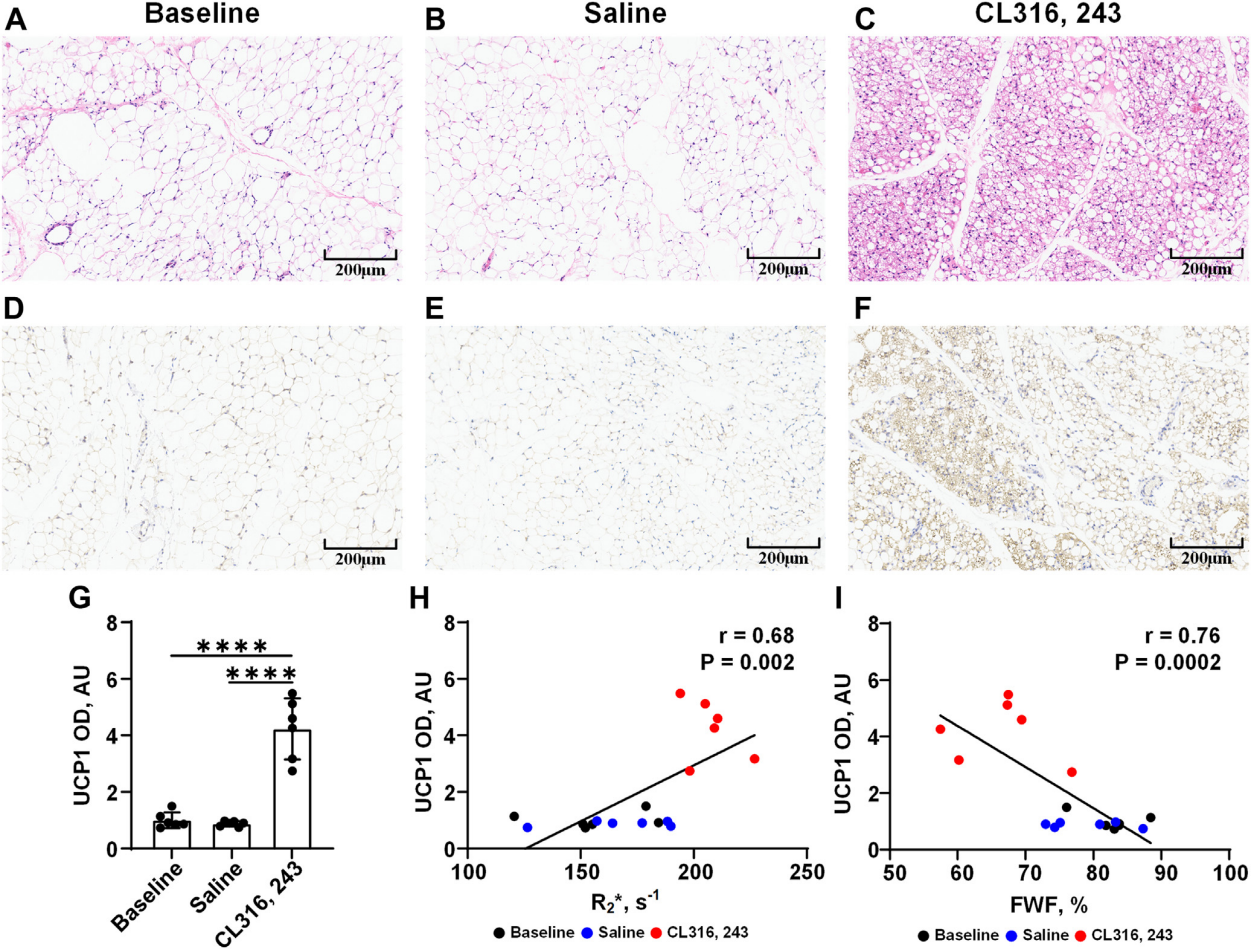
Histological changes in iWAT before and after CL administration for 7 days. Representative H & E staining of the iWAT of mice in the baseline (A), saline (B), and CL groups (C), respectively. Scale represents 200 μm. Representative IHC staining of the iWAT of mice in the baseline (D), saline (E), and CL (F) groups, respectively. Scale represents 200 μm. G: Comparison of the absorbance of UCP1 in iWAT of mice among the baseline, saline, and CL groups. Data are presented as mean ± SD. ∗∗∗∗*P* < 0.0001. H: Correlation between R_2_∗ value and UCP1 expression. I: Correlation between FWF and UCP1 expression. AU, arbitrary unit.

H&E and IHC (UCP1) staining was performed on iWAT. As seen in the H&E staining (Fig. 5A–C), after 7 days of repeated CL stimulation, mice had a significant increase in multilocular small lipid droplet adipocytes compared with the baseline and saline groups, which indicates the generation of brown adipocyte-like cells, referred to as beige adipogenesis. Both the baseline group and the saline group showed single large fat droplet adipocytes. The browning of iWAT was also confirmed by UCP1-IHC staining (Fig. 5D–F). Increased UCP1 expression in iWAT was found after CL stimulation for 7 days compared with the baseline and saline groups.

Quantitative analysis of absorbance from IHC showed that the absorbance of UCP1 in iWAT was low at baseline (1.0 ± 0.3 arbitrary unit [AU] and saline group (0.9 ± 0.1 AU) and increased significantly in the CL group (4.2 ± 1.1 AU) (both with *P* < 0.0001; Fig. 5G). The absorbance of UCP1 in iWAT was strongly correlated with both R_2_∗ value (r = 0.68, *P* = 0.002; Fig. 5H) and FWF (r = 0.76, *P* = 0.0002; Fig. 5I).

### Gene expression analysis detects the browning of iWAT

**Fig. 6:**
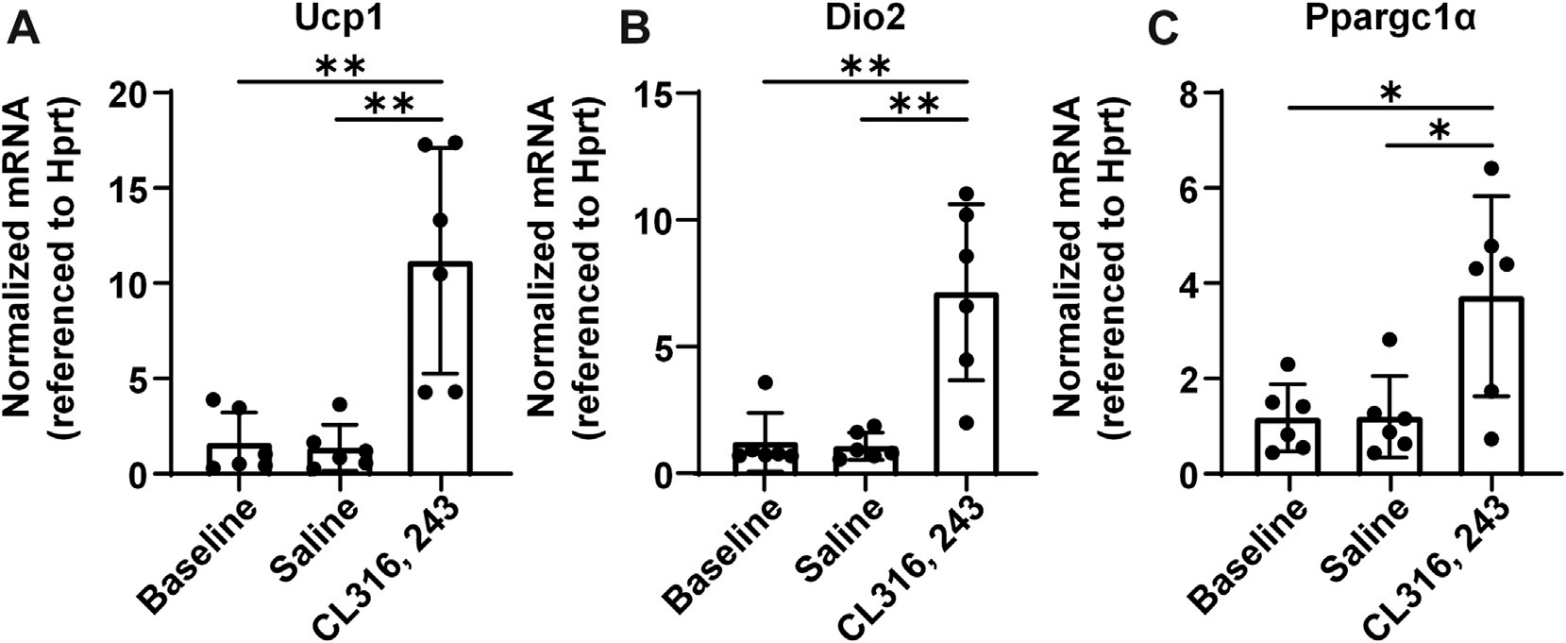
Thermogenesis-related gene expression in iWAT before and after CL administration for 7 days. Comparison of the thermogenesis-related gene expression of Ucp1 (A), Dio2 (B), and Ppargc1α (C) genes in iWAT of mice among the baseline, saline, and CL groups. Quantitative PCR data are normalized to Hprt mRNA and presented as mean ± SD. ∗*P* < 0.05, ∗∗*P* < 0.01.

As shown in Fig. 6, at the level of gene transcription, the expressions of related thermogenic genes (such as UCP1, Dio2, and Ppargc1α) in the CL-treated group were increased significantly compared with the control and saline groups.

### The in vivo R_2_∗ value of iWAT is highly correlated with ex vivo iron content

**Fig. 7:**
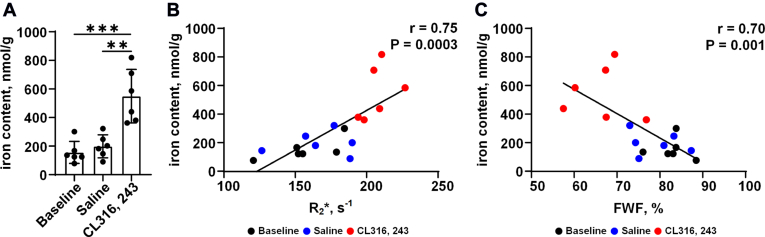
Iron content changes in iWAT before and after CL administration for 7 days. A: Comparison of the ex vivo iron content of iWAT of mice among the baseline, saline, and CL groups. B: Correlation between in vivo R_2_∗ value and ex vivo iron content. C: Correlation between FWF and ex vivo iron content. Data are presented as mean ± SD. ∗∗*P* < 0.01, ∗∗∗*P* < 0.001.

The results of the ex vivo iron content assay showed that the total iron content was significantly higher in the CL group than in the baseline and saline groups (baseline vs. CL: 155.4 ± 77.1 nmol/g vs. 549.6 ± 187.1 nmol/g, *P* < 0.001; saline vs. CL: 198.4 ± 80.6 nmol/g vs. 549.6 ± 187.1 nmol/g, *P* < 0.01; Fig. 7A). Pearson's correlation analysis showed the in vivo R_2_∗ values of iWAT correlated well with ex vivo the total iron content (r = 0.75, *P* = 0.0003; Fig. 7B). The FWF of iWAT also had a good correlation with ex vivo the total iron content (r = 0.70, *P* = 0.001; Fig. 7C).

### The R_2_∗ value in iWAT was strongly and inversely correlated with its FWF

**Fig. 8:**
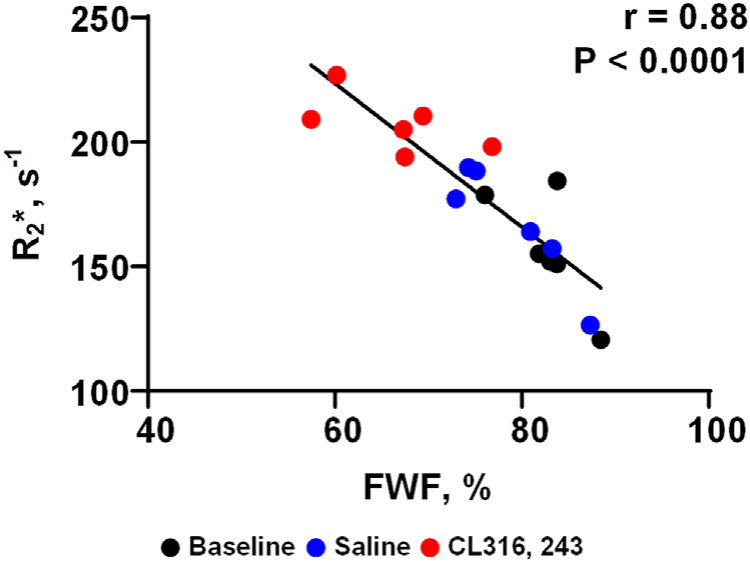
The correlation between R_2_∗ and FWF in iWAT. R_2_∗ values were negatively correlated with the FWF in iWAT.

The correlation analysis showed that the R_2_∗ value of iWAT was strongly correlated with its FWF (r = 0.88, *P* < 0.0001; Fig. 8).

## Discussion

This study investigated the browning of iWAT induced by a relatively long-term CL drug stimulation in mice and quantified the changes in R_2_∗, FWF, UCP1 expression, and iron content. We observed a significant increase in R_2_∗ and a decrease in FWF in iWAT after 7 days of CL injection and found that the UCP1 expression level of iWAT was strongly correlated with R_2_∗ and FWF. We also observed that the in vivo R_2_∗ value was highly correlated with the ex vivo iron content. Importantly, the R_2_∗ value of iWAT was strongly and inversely correlated with its FWF. These results suggested that R_2_∗ is a potential imaging biomarker for the assessment of WAT browning.

R_2_∗ is positively correlated with iron content (26). We observed that there are background R_2_∗ signals in the baseline and saline groups. First, every tissue has its intrinsic R_2_∗ regardless of the presence of iron or UCP1 given that R_2_∗ (or 1/T_2_∗) is one of the fundamental relaxation properties of MRI similar to R_1_ (or 1/T_1_) and R_2_ (or 1/T_2_). Second, the iWAT contains a certain number of mitochondria (27) and vascular systems (28, 29) containing hemoglobin and iron contents. These explain why there are background R_2_∗ signals in the baseline and saline groups.

In this study, the iWAT R_2_∗ was significantly increased in mice injected with CL for 7 days compared with the baseline group, whereas there was no significant change in R_2_∗ in mice with saline injection. The results of this study suggested that the iron content of iWAT increases during the browning process. This finding was also confirmed by ex vivo iron determination. In a previous study, after 5 days of CL injection, a significant increase in iWAT iron content was observed in mice (21). Many studies have shown that iron is essential for adipocyte differentiation and thermogenesis (27, 30, 31). Mitochondrial iron content enhances the activity of iron-containing electron transport chain complexes and enzymes (32, 33), thereby enhancing cellular respiratory activity. In contrast, two studies that selectively deleted transferrin receptor 1 in adipocytes to block iron entry found that iron depletion reduced mitochondrial biogenesis and impaired thermogenesis in adipocytes (34, 35). So, iron deficiency impairs the formation of beige adipocytes and reduces the thermogenic capacity of beige adipocytes. The basic mechanism of how iron metabolism regulates beige adipogenesis has been reported (16). Activation of the ADRB3 triggers two coordinated modes of iron regulation during WAT browning. One mode involves intracellular iron regulation in adipocytes, where the iron regulatory protein binds to the iron response element within the adipocytes. The second mode relies on systemic iron mobilization, which is influenced by body oxygen levels or acts independently. More specifically, the stimulation of ADRB3 to promote beige fat formation requires iron-mediated activation of iron regulatory protein/iron response element signaling. This activation leads to a series of changes including acute hypoxia in the kidney, stress erythropoiesis in the spleen, and downregulation of hepcidin in the liver, triggering the release of iron stored in organs such as the liver and spleen, which is then redistributed to beige fat for mitochondrial biogenesis. Furthermore, we observed a strong positive correlation between in vivo R_2_∗ of iWAT and ex vivo iron content of iWAT, suggesting R_2_∗ as a potential imaging biomarker for the assessment of WAT browning.

The FWF of iWAT decreased after 7 days of CL injection. Tomaszewski *et al.* (29) observed a significant decrease in the FWF of the iWAT after 2 weeks of repeated CL injections in mice, which is consistent with the results observed in this study. In response to chronic CL stimulation, the FWF of the iWAT decreased, likely because of the enhancement of mitochondrial β-oxidation and the significant increase in lipid oxidation during the browning of iWAT. The thermogenic process in activated iWAT is associated with UCP1. Repeated CL stimulation for 7 days resulted in a significant increase in UCP1 expression levels in iWAT. In addition, there was a good correlation between in vivo FWF and ex vivo UCP1 expression levels of iWAT. This suggests that FWF can detect thermogenic responses after WAT browning.

The close correlation between quantitative imaging parameters and actual biological effects is crucial for identifying a useful imaging biomarker. UCP1 is a key protein that regulates thermogenesis in adipocytes (36, 37). In this study, the positive correlation between the UCP1 expression level and the R_2_∗ of iWAT suggests that R_2_∗ may reflect the browning process of WAT. There is no doubt that R_2_∗ has less specificity than UCP1 staining. The lack of correlation within the CL-treated mice is because the intersubject variation surpassed the relatively small changes of UCP1, yielding a nonsignificant correlation with a small group. In addition, UCP1 quantification as a histological method has limitations because of its potential sampling error and bias, so it is challenging to reliably capture the small intragroup UCP1 changes.

What’s more, previous studies have reported that changes in FWF can be used to characterize WAT browning (28, 29). FWF, a lipid composition index of WAT browning given that FWF reduces during browning. The FWF before and after the browning of iWAT showed a strong correlation with R_2_∗ values in this study, which gives a clue that R_2_∗ may serve as an independent imaging biomarker for WAT browning. Mitochondrial iron content enhances the activity of iron-containing electron transport chain complexes and enzymes, thereby enhancing cellular respiratory activity (32, 33). In contrast, selectively deleted transferrin receptor 1 in adipocytes to block iron entry found that iron depletion reduced mitochondrial biogenesis and impaired thermogenesis in adipocytes (34, 35). So, iron deficiency impairs the formation of beige adipocytes and reduces the thermogenic capacity of beige adipocytes. In conclusion, for specific circumstances involving iron-regulated pathways and interventions for WAT browning, R_2_∗ may be a more valuable and specific biomarker than FWF.

There are, however, some limitations to this study. First, the sample size is rather small, and only male mice were included in the study. Second, only two time points were investigated, and it is unknown when is the earliest time for detectable WAT browning. Further studies at multiple time points are needed to monitor the full evolution of WAT browning. What’s more, combining multiparametric MRI parameters will likely further improve the correlation with UCP1. However, such radiomics studies typically utilizing a variety of imaging biomarkers, big data, and large sample sizes deserve comprehensive design for the development of convincing mathematical or artificial-intelligent models. Finally, this study is still in the preclinical stage. In the future, it is necessary to apply these findings to clinical practice, which may help the screening of drugs and the adjustment of strategies for inducing WAT browning in humans.

## Conclusion

In conclusion, R_2_∗ has been demonstrated as a promising quantitative indicator for assessing WAT browning, a potential strategy for combating obesity and metabolic-related diseases. Future studies are necessary with large sample sizes in preclinical settings as well as in patients to validate the translation of this imaging method to clinical applications.

## Data availability

The data that support the findings of this study are available from the corresponding author upon reasonable request.

## Conflict of interest

The authors declare that they have no conflicts of interest with the contents of this article.
